# Sex-Gender Comparisons in Comorbidities of Children and Adolescents With High-Functioning Autism Spectrum Disorder

**DOI:** 10.3389/fpsyt.2019.00159

**Published:** 2019-03-26

**Authors:** Lucia Margari, Roberto Palumbi, Antonia Peschechera, Francesco Craig, Concetta de Giambattista, Patrizia Ventura, Francesco Margari

**Affiliations:** ^1^Basic Medical Sciences Neuroscience and Sense Organs Department, University of Bari Aldo Moro, Bari, Italy; ^2^Unit for Severe Disabilities in Developmental Age and Young Adults, Developmental Neurology and Neurorehabilitation, Scientific Institute IRCCS E. Medea, Brindisi, Italy

**Keywords:** high-functioning, autism spectrum disorder, psychopathological comorbidities, gender distribution, anorexia nervosa, ADHD, anxiety disorders, mood disorders

## Abstract

Over the last few years, new studies focused their attention on the gender-related features in high-functioning autism spectrum disorder (HFA), often leading to controversial results. Another interesting aspect of these subtype of patients is linked to the complexity of clinical presentation, where besides core symptoms, other co-occurrence disorders may complicate the diagnostic evaluation. Therefore, we retrospectively studied 159 HFA patients, male and female, investigating their comorbidities and to find any gender difference. For each patient, were evaluated the presence/absence, type and gender distribution of psychopathological comorbidities, according to DSM-5 diagnostic criteria. The total sample was divided in 100 male and 59 female patients, age and intelligence quotient matched. In our sample, the psychiatric comorbidities observed were Attention Deficit Hyperactivity Disorder, Anxiety Disorders, Depressive Disorders, Bipolar Disorder, Obsessive-Compulsive Disorder, and Anorexia Nervosa. No statistical significant differences were found between male and female HFA patients comorbidities except for Anorexia Nervosa. In both male and female patients, attention deficit and hyperactivity disorder and anxiety disorders were found in high percentage. In conclusion, our investigation showed that a statistical significant difference of comorbidity between male and female HFA patients was found only for AN diagnosis. However, the question about the distinction between female and male HFA patients remains quite interesting and an open area of research for future studies.

## Introduction

It is well-known that in Autism Spectrum Disorder (ASD), males are over-represented than females, with an average gender ratio of 4.3 males to 1 female (1). Many theories have emerged in order to explain this difference in gender distribution but no one explanation appears to be conclusive (2–5). Females are different from males both in core symptoms and in comorbidity; this probably causes the difficulty in their detection. Data show that the gender ratio M:F is intelligence quotient (IQ)-related, varying from 5.75:1 in cognitively high-functioning children (HF; full-scale IQ higher than 70) to 1.9:1 in low-functioning children (LF; full-scale IQ lower than 70) (5–9).

The definition of High Functioning Autism Spectrum Disorder (HFA) refers to the category of ASD “without cognitive impairment” specified by the Diagnostic and Statistical Manual of Mental Disorders—Fifth Edition (10).

This variance shows that HF females have been the most difficult to detect, probably because of milder symptom presentation (11–13) or methodological bias (ex. lack of specific diagnostic tools) (14–16). With increased agreement on the definition of ASD and improvement in case detection, recent studies are identifying more HF females (17). Beyond the sex differences in phenotype, males and females could differ in coexisting psychopathology (5) so there has been an emergence of research focusing on gender differences in ASD regarding comorbidity. The most common practice to date for identifying comorbid psychopathologies had been the use of the Diagnostic and Statistical Manual of Mental Disorders (DSM) criteria. Literature on comorbid psychopathologies, based on the new edition of DSM criteria (DSM-5), is beginning to develop, even if with mixed findings, probably caused by different methodologies (related to stratification for IQ and age) and by small samples.

A report described that boys with ASD are more likely to experience externalizing disorders such as ADHD and oppositional defiant disorder (18), above all in childhood, while it is reported that girls with ASD may be at especially high risk for internalizing psychopathology (8).

Moreover, studies have shown more hyperactive behaviors in HF boys than girls with ASD (19), while no gender differences in LF children (20–23) were found.

Nevertheless, differences also emerged with respect to internalizing disorders as HF girls with ASD evidenced significant anxiety symptoms compared to boys, mainly in adolescence (8), suggesting that the typical gender developmental trajectory of anxiety may be present. However, it might be quite difficult to distinguish if anxiety symptoms or repetitive behaviors would belong to the core ASD symptoms or might be signs of a comorbid condition with an anxiety and/or an obsessive-compulsive disorder (24). In the same way as for anxiety symptoms, other affective disorders, such as depressive disorders, are reported in childhood and at levels dramatically higher in adolescent girls (16, 19, 25). Lastly, in ASD patients are largely reported abnormal eating behaviors and/or conducts (26).

However, sex differences in HFA comorbidities is still poorly understood. Therefore, the aim of this retrospective study was to investigate whether male and female HFA patients might develop specific comorbidities phenotype, using well-defined samples regarding factors like age and IQ.

## Materials and Methods

This retrospective study included children and adolescents admitted to the Child and Adolescent Neuropsychiatry Unit between April 2016 and May 2018 and diagnosed with HF ASD, according to DSM-5 diagnostic criteria. For each patient, were evaluated the presence/absence, type and gender distribution of psychopathological comorbidities. All participants were drug-naïve.

All demographic and clinical variables were subjected to statistical analysis. Descriptive analysis was conducted for sociodemographic and clinical features. Quantitative variables (IQ and age) were presented as mean ± standard deviation (SD); qualitative variables (psychopathological comorbidities and ASD severity level) were expressed as percentages. The chi-square test (χ2) was used to compare qualitative variables between male and female with ASD. The χ2 enabled us to compare observed and expected frequencies of dichotomous variables objectively. Statistical significance, in this case, is due to the difference between observed and expected frequencies. Both groups were compared with the independent sample *t* test for comparison of age and IQ mean score. All the statistical analyses were considered significant with a *p*-value equal or lower than 0.05. For statistical processing, we used the Statistical Package for Social Science version 20.0.

## Results

The sample included 159 patients with HF ASD diagnosis. Table 1 summarizes patients demographic and clinical data. The total sample included 100 males (mean age: 9.91 ± 4 DS) and 59 females (mean age: 10.97 ± 4.7 DS). No significant statistical differences for age and Intelligent Quotient were found between male and female patients. In 44% of male patients at least one comorbidity was diagnosed, while, in female patients, 72.8% presented at least one comorbidity.

**Table 1:** Demographic and clinical characteristics of ASD patients.

	**ASD male (*n = 100*)**	***ASD female* (*n = 59*)**	***p***
Age (mean ±*SD*)	9.91 ± 4	10.97 ± 4.7	0.140
IQ (mean ±*SD*)	108 ± 16	104 ± 18	0.137
**ASD SEVERITY LEVEL**
Level 1	84	46	0.341
Level 2	16	13	0.341

*ASD, autism spectrum disorder; IQ, intelligence quotient; SD, standard deviation*.

In both male and female patients, ADHD and Anxiety Disorders were the most frequent diagnoses. Anorexia Nervosa diagnosis resulted more frequent among female patients, with a statistical significant difference (*p* = 0.04) (Table 2).

**Table 2:** Comparison of ASD psychopathological comorbidities in male and female patients.

**Neuropsychiatric diagnosis**	**ASD male (%)**	**ASD female (%)**	***p***
Attention deficit hyperactivity disorder	32	42.7	0.156
Anxiety disorders	18	25.4	0.265
Depressive disorders	6	13.5	0.104
Bipolar disorder	4	3.4	0.845
Obsessive-Compulsive disorder	5	0	0.081
Anorexia nervosa	1	6.8	**0.004**

*ASD, autism spectrum disorder. Bold values indicate statistical significant p-value*.

## Discussion

It is well-established that the most difficult challenge in ASD diagnostic process is based on the recognition and discrimination of the frequent co-occurrence of this disorder with other conditions, such as other neurodevelopment and/or psychiatric disorders. In fact, core symptoms of ASD often mask psychiatric comorbid symptoms and viceversa (27–29). Furthermore, there are potential similarities of how the symptoms of these disorders appear (24).

The purpose of this study was to investigate HF ASD psychopathological comorbidities between male and female patients. In our sample, high rates of comorbidities were found. Moreover, 55% of the total sample presented at least one comorbidity, with a higher rate in females (72.8%) than males (44%). This finding about the high prevalence of comorbidities in ASD is in accordance with literature data (28). The difference between the comorbidities rates between male and female patients in our sample may be a statistical bias due to the disproportion of the sample sizes. Eventually larger sample studies would contribute to clarify if this percentage difference of comorbidity exists or not. Moreover, in female HFA patients, it is more difficult to recognize psychiatric comorbidities for several reasons. First, diagnostic criteria and/or tools are mainly male-targeted complicating their detection; moreover, females less refer to clinical attention since their impairment is even less clear than in male patients.

In our sample, the psychiatric comorbidities observed (Table 2) were ADHD, Anxiety Disorders (AD), Depressive Disorders (DD), Bipolar Disorder (BD), Obsessive-Compulsive Disorder (OCD), and Anorexia Nervosa (AN). No statistical significant differences were found between male and female HFA patients comorbidities except for AN.

The most frequent comorbidity was ADHD with no statistical significant difference between male and female subjects. Over the years, and lastly after the publication of DSM-5, the overlap between ASD and ADHD has been supported by an increasing number of studies (28, 30–35). A recent study estimated that the prevalence of the co-occurring of ASD and ADHD is about 37–85% of children with ASD (35), supporting the hypothesis of a common neurobiological pathogenesis (35, 36). Moreover, comparing male with female, we found that ADHD with “Predominantly inattention presentation” prevails on female subgroup; while in male subgroup “Combined presentation” is the most frequent clinical presentation. Potentially, associated difficulties commonly occurring in HFA, like externalizing/disruptive behaviors, could differ between boys and girls prompting gender differences in the clinical referral. Girls without externalizing behaviors/hyperactivity could be overlooked for assessment and educational support despite largely similar cognitive, academic and behavioral profiles to boys (37, 38).

In our sample, Anxiety Disorders was found in high percentage of both male and female patients, without any statistical significant difference. In a recent review, Tarazi et al. (39) found that people with HFA experience more anxiety symptoms (such as tension, apprehension, panic, attention deficits) compared to other ASD patients. It is suggested that, in this subtype of patients, anxiety is more likely related to their inability to face social interactions, changing in daily routines or modulation of their emotional experiences.

In our sample, Depressive and Bipolar Disorders appeared to be less frequent than other comorbidities. This finding is probably due to the typical adolescent onset of these mood disorders in ASD, and the mean age of our sample was about 10 years old (8, 16).

In our total sample, OCD diagnosis was found in 3%; this finding probably is understandable considering that OCD could appear particularly difficult to identify in the context of an ASD because of their potential similarities only in five male patients. Moreover, OCD was diagnosed only in five male patients; this may be probably explained by the fact that restrictive interests and ritualistic behaviors in female HFA patients appear to be more socially acceptable (40).

Anorexia Nervosa was more frequent among female subjects with a statistical significant difference. This result reflects the epidemiology of AN, considering that this disorder predominantly affects female patients in early adolescence (41). It is suggested that both genetic and psychosocial risk factors may contribute to this gender difference of AN prevalence. Firstly, during puberty, ovarian hormones are directly involved in genetic effects as transcriptional mediators of neural expression of transmission systems disrupted in eating disorder (e.g., serotoninergic system) (42, 43). Secondly, young girls are more exposed than males to sociocultural factors that may contribute to increase the risk of AN (e.g., pressure about weight and body image, thinness and cultural model) (43, 44). Moreover, it is well-known how early testosterone exposure has a huge impact on brain plasticity and organization, and this effect may contribute to protect male subjects from developing an eating disorder (45–48). The association between AN and ASD was firstly described in 1980s when Gillberg observed that three male autistic patients had a familiar history for AN (49, 50). The author hypothesized that common factors may contribute to develop autism in young boys and AN in female relatives. On the other hand, dysfunctional eating behaviors (such as restrictive and/or limited food intake or repertoire) represents one of the main clinical aspects of ASD (51). Moreover, it is reported that abnormal eating features may be precursors of AN in patients with ASD (52). Nevertheless, patients affected by AN share clinical similarity with ASD (e.g., set-shifting deficits, reduced cognitive flexibility, deficits in emotion recognition, and social cognition) and some of these features might be potential risk factors to develop AN (41). It is suggested that these aspects may be explained by a possible neuropsychological overlap between the two disorders (deficit in central coherence, theory of mind and executive functions theories) (53). A recent study investigated gray matter volumetric aspects in female patients affected by AN and autistic traits. The results showed that higher autistic traits correlate with volumetric alterations of brain regions involved in the social cognition, supporting a valuable link of the association between the two disorders (54). In addition, the overlap between ASD and AN may be supported by other common neurobiological basis, including the involvement of the dopaminergic system. Variations in dopamine transporters and dopamine receptors gene (e.g., DAT1 and DRD4) are strongly linked to ASD (55–58). Nevertheless, it is known that dopamine is one the crucial neuromediator involved in feeding behavior, distortion of body image perception (59, 60). In fact, mesolimbic dopamine pathways play a crucial role in food reward, anticipation of food and social recognition mechanisms (61, 62). Moreover, specific genotype of DRD4 (DRD4 7R/R), leading to a reduced expression of the receptor, appears to be strongly linked to the risk of AN (59, 63). However, the complete neurobiological basis of the overlap between AN and ASD are quite uncertain and still not defined.

In conclusion, our investigation showed that a statistical significant difference of comorbidity between male and female HFA patients was found only for AN diagnosis. However, the question about the distinction between female and male HFA patients remains quite interesting and open. Furthermore, female HFA patients are often overlooked, misdiagnosed and underestimated; therefore, this retrospective study might be preliminary for future and larger-sampled research on this category of patients.

## Ethics Statement

For this study, an ethical review process by the Local Ethics Committee of Azienda Ospedaliero-Universitaria Policlinico di Bari (Italy) was not required, since all the procedures within the study assessment are included in the diagnostic protocol of our Child and Adolescence Neuropsychiatry Unit. All the participants were recruited after obtaining a written informed consent by their parents.

## Author Contributions

LM, PV, and FM designed and supervised the study. RP, AP, and CdG contributed to the recruitment and the revision of the medical records. FC performed the statistical analyses. All authors equally contributed to the draft of the manuscript.

### Conflict of Interest Statement

The authors declare that the research was conducted in the absence of any commercial or financial relationships that could be construed as a potential conflict of interest.
